# The Effects of Tryptophan on Everyday Interpersonal Encounters and Social Cognitions in Individuals with a Family History of Depression

**DOI:** 10.1093/ijnp/pyv012

**Published:** 2015-03-23

**Authors:** Koen Hogenelst, Robert A. Schoevers, Marije aan het Rot

**Affiliations:** Department of Psychology (Mr Hogenelst and Dr aan het Rot), School of Behavioral and Cognitive Neurosciences (Mr Hogenelst and Dr aan het Rot), and University Medical Centre Groningen, Department of Psychiatry (Dr Schoevers), University of Groningen, Groningen, The Netherlands.

**Keywords:** serotonin, social interaction, mood, major depressive disorder, dysfunctional attitudes

## Abstract

**Background:**

Individuals with a family history of depression show subtle abnormalities in the processing of social stimuli. This could negatively affect their interpersonal functioning and contribute to their depression risk. Repeated administration of the serotonin precursor tryptophan has previously been shown to increase agreeable behavior and reduce quarrelsome behavior in irritable people, who are also considered at risk for depression.

**Methods:**

To examine the effects of tryptophan on social functioning in individuals with a family history of depression, 40 men and women with at least one first-degree relative with depression received tryptophan (1g three times a day) and placebo for 14 days each in a double-blind crossover design and recorded their social behavior and mood during everyday interpersonal encounters. Participants also provided daily ratings of their positive and negative cognitions concerning their social functioning.

**Results:**

Tryptophan improved mood. Unexpectedly, tryptophan increased quarrelsome behavior and reduced agreeable behavior, specifically during interactions at home. The behavioral effects of tryptophan were not moderated by mood or by the interaction partner. Negative social cognitions were lower when tryptophan was given second and lower during placebo when placebo was given second.

**Conclusion:**

Overall, tryptophan may not alter social behavior in individuals with a family history of depression as it does in irritable people. However, the behavioral effects of tryptophan at home might be seen as a way for individuals with a family history of depression to achieve more control. Over time, this may positively influence the way they feel and think about themselves in a social context.

## Introduction

Individuals with major depressive disorder (MDD) often show impairments in their interpersonal functioning (Hirschfeld et al., 2000; Hames et al., 2013). Depression has been associated with reduced cooperation (Hokanson et al., 1980) and elevated irritability and hostility (Perlis et al., 2005). These behavioral patterns are thought to produce interpersonal problems that in turn are associated with the maintenance and severity of MDD (Hames et al., 2013).

Experiencing interpersonal problems may also affect the way MDD patients think about themselves in relation to others. Depression is often accompanied by dysfunctional cognitions (Beck, 2008), which regularly revolve around interpersonal themes (Joiner, 2002). MDD patients tend to evaluate their social environment in a negative way (Beck, 2008) and often think they are regarded unfavorably by others (Youngren and Lewinsohn, 1980). Dysfunctional cognitions are also thought to contribute to the course of MDD, and reducing dysfunctional cognitions during treatment has been suggested to contribute to treatment efficacy (e.g., DeRubeis et al., 1990).

Having a first-degree relative with MDD significantly increases the likelihood of MDD (Sullivan et al., 2000). As interpersonal stress, rejection, and low social support are risk factors for MDD (Hirschfeld et al., 2000; Hames et al., 2013), it is plausible that a family history of depression (FH+) is associated with difficulties in social functioning. This has indeed been found (Watters et al., 2013). Poor social functioning in FH+ individuals may be due to the existence of subtle impairments in the processing of emotional stimuli (Mannie et al., 2007). This can be a source of irritability, which is also common in FH+ individuals (Lauer et al., 1997). As irritability can elicit negative reactions in others (Moskowitz, 2010), it can cause interpersonal stress or rejection by others and a loss of social support. Thus, impairments in the processing of emotional stimuli may negatively affect the social interactions of FH+ individuals and thus contribute to their MDD risk.

Serotonin remains an important target in depression research and treatment (Albert et al., 2012). Alterations in the brain serotonin system, caused by (epi)genetic factors and stressful experiences, are thought to lower its capacity to regulate mood, thereby predisposing individuals to MDD (Albert et al., 2012; Talati et al., 2013). Serotonin normally helps regulate mood by modulating emotional responses to environmental stimuli (Young, 2013; Harmer and Cowen, 2013). Environmental influences are often of a social nature (Kiser et al., 2012). In this context, it is interesting that the emotional processing impairments that have been observed in FH+ individuals can be exacerbated both by negative mood states (Taylor and Ingram, 1999) and by a temporary lowering of brain serotonin levels (van der Veen et al., 2007; Feder et al., 2011).

Few experimental studies have assessed the role of serotonin in the social functioning of MDD patients. Treatment with a serotonergic antidepressant has been found to reduce anger attacks (Fava et al., 1997) and increase extraversion (Tang et al., 2009). In another treatment study, early reductions in subjective hostility predicted later antidepressant response (Farabaugh et al., 2010). These studies provide limited evidence that serotonergic antidepressants may positively influence the social functioning of MDD patients and that this might contribute to clinical outcome.

While antidepressants have immediate synaptic effects, several weeks of treatment are required for therapeutic effectiveness (Frazer and Benmansour, 2002). According to Harmer et al. (2013), antidepressants acutely improve the processing of socio-emotional stimuli. Over time, this remediates negative perceptual biases, and ultimately this contributes to reduced depressive symptoms. Complementary to this idea, Young et al. (2014) proposed that antidepressants might work by promoting agreeable behavior and reducing quarrelsome behavior. Over time, these behavioral changes may elicit similar changes in social interaction partners. The cumulative effects of more positive interactions may result in gradual mood improvement and may contribute to the clinical effectiveness of antidepressants. Overall, antidepressants may improve social functioning both directly (Young et al., 2014) and indirectly (Harmer and Cowen, 2013).

It is possible that improvements in social functioning in MDD patients during antidepressant treatment can occur independently of a reduction in depressive symptoms. In line with this idea, antidepressant treatment has been found to improve aspects of social functioning in healthy volunteers. For example, Knutson et al. (1998) observed a decrease in subjective irritability and an increase in affiliative behavior during a dyadic puzzle task. Tse and Bond (2002) observed an increase in cooperative behavior during a mixed-motive game. As these studies involved computer tasks or standardized social interactions, single observations, and artificial settings, their relevance to actual social functioning may be limited.

However, other studies have used ecological momentary assessment (EMA) to investigate the effects of increased brain serotonin on social interactions (Moskowitz et al., 2001; aan het Rot et al., 2006). As EMA encompasses the intensive, repeated assessment of people’s thoughts, feelings, and behaviors in everyday situations, the ecological validity of EMA data is considered high (Moskowitz and Young, 2006). Moskowitz et al. (2001) found that healthy volunteers reported less quarrelsomeness when taking tryptophan, the amino acid precursor to serotonin, compared with when taking placebo. No significant mood change was observed. aan het Rot et al. (2006) subsequently recruited healthy individuals with high trait irritability. Tryptophan improved mood and decreased quarrelsomeness. Further, tryptophan increased agreeableness, an effect that was independent of the observed change in mood. The study by aan het Rot et al. (2006) in particular provides evidence for the idea that increasing serotonin may promote more positive social interactions directly (cf. Young et al., 2014 discussed above).

Similar to high trait irritable individuals (Conner et al., 2003), FH+ individuals constitute a population at high risk for MDD (Sullivan et al., 2000). Studies in FH+ individuals may inform about the role of serotonin in social functioning in MDD even more than studies conducted with high trait irritable individuals (cf. aan het Rot et al., 2006). Since many FH+ individuals have never used psychotropic medications, the effects of tryptophan can be studied without needing to take depressive symptoms and antidepressant use into account.

The present EMA study assessed the effects of tryptophan on the social interactions of FH+ individuals. As we aimed to extend previous findings, we hypothesized that tryptophan would decrease quarrelsomeness, increase agreeableness, and improve mood. We also aimed to assess the effects of tryptophan on social cognitions. Specifically, we hypothesized that when taking tryptophan individuals would report more positive and fewer negative thoughts about themselves in relation to others. Social cognitions were assessed at the end of each day of EMA, so we could determine whether the effects of tryptophan on interpersonal functioning would extend beyond the level of individual interactions.

## Methods

### Participants

The study was approved by the Medical Ethics Committee of the University Medical Centre Groningen and executed in accordance with the Declaration of Helsinki. All participants signed an informed consent form after the procedures of the study had been explained to them in writing and verbally. They received 100 euro for their participation.

Men and women could participate if they had at least one first-degree family member with MDD and met the following inclusion criteria: age 18 to 65 years, no current or past DSM-IV mood disorder including MDD, no other current DSM-IV Axis-1 disorder, no current major medical illness, no current use of psychotropic medications, and no contraindication for the use of tryptophan. Participants were screened using the Structured Clinical Interview for DSM-IV Axis I disorders (First et al., 2002) and asked about their psychiatric family history using the method described by Andreasen et al. (1977).

Full details of the screening phase can be found in the supplementary information. Briefly, 42 participants started the study. One participant dropped out after 5 days and one participant admitted after the study to nonadherence to the EMA instructions. The results are described for 40 participants (13 men, 27 women).

### Treatment

In a double-blind crossover design, all participants took two 500-mg l-tryptophan (Cell Care, Putten, The Netherlands) or identical placebo capsules 3 times a day for 14 days. Treatment order was counterbalanced within gender. We implemented a 7-day inter-treatment interval. The 3-g daily dose of tryptophan was identical to the daily dose used in previous studies (Moskowitz et al., 2001; aan het Rot et al., 2006).

### EMA

Participants used EMA to record their social interactions throughout the 2 treatment periods. Standardized forms asked participants about the broad social context of each interaction and how they behaved and felt. Participants were instructed to record alcohol use in the 3 hours before or during the interaction and to not complete any forms after illicit drug use.

#### Measurement of Behavior

We used a Dutch translation of the Social Behavior Inventory (SBI) (Moskowitz, 1994), previously found to differ minimally in the interpretation of the 46 behavioral items (aan het Rot et al., 2013). These items are used to assess agreeableness (e.g., “I exchanged pleasantries”), quarrelsomeness (e.g., “I confronted the other about something I did not like”), dominance (e.g., “I assigned someone to a task”), or submissiveness (e.g. “I gave in”). One item assesses both dominance and quarrelsomeness (“I criticized the other”) and one item assesses both agreeableness and submissiveness (“I went along with the other”). The original SBI has been shown to provide valid and reliable scores of each dimension of social behavior (Moskowitz, 1994; Moskowitz and Sadikaj, 2012).

Participants were instructed to mark all behaviors they engaged in during a social interaction. The 46 SBI items were divided over 4 forms, rotated on a daily basis to prevent participants from marking the same behaviors for every interaction. On each form, each dimension of behavior was represented by 3 items. Ipsatized behavior scores were calculated using the method previously described by Moskowitz et al. (2001) and aan het Rot et al. (2006). These ipsatized scores reflect the extent to which behaviors pertaining to a specific dimension are checked relative to a participant’s overall rate of behavior checking. As people tend to check quarrelsome and submissive behaviors less often than agreeable and dominant behaviors, the ipsatized scores for quarrelsome and submissive behaviors tend to be low, and often negative.

#### Mood Measurement

A list of affect adjectives was used as a proxy for mood state (Diener and Emmons, 1984). On each form these were rated on a scale from 0 (not at all) to 6 (extremely). Event-level means were calculated for positive affect (PA) (e.g., happy, pleased, joyful) and negative affect (NA) (e.g., worried/anxious, angry/hostile, depressed) separately.

### Measurement of Daily Social Cognitions

At the end of each day, participants were asked to indicate on a scale from 0 (not at all) to 6 (all the time) to what extent they had certain negative thoughts (e.g., “No one understands me”) and certain positive thoughts (e.g., “I have a good way with others”). The list of social cognitions contained 12 items (see supplemental Information for more information).

A factor analysis of the data obtained in the placebo phase indicated a 3-factor structure with all 6 positive cognitions loading on 1 factor, 5 negative cognitions loading on the second factor, and 1 negative cognition loading on a third factor (“I am a social failure”). Internal consistency measured by calculating Cronbach’s alpha was excellent for the items representing positive cognitions (α = 0.95) and acceptable for the negative cognitions loading on the second factor (α = 0.60). Thus, day-level means were calculated for the 6 positive cognitions and for 5 of the 6 negative cognitions.

### Procedure

The day before the first EMA day, participants were instructed extensively about the method. They also completed the Quick Inventory of Depressive Symptomatology (QIDS-SR) (Rush et al., 2003) and the Revised Leiden Index of Depression Severity (LEIDS-R) (Van der Does, 2002). For the 28 assessment days, participants received 2 packages, each with 14 preaddressed stamped envelopes. Each envelope contained 10 social interaction forms and the tryptophan or placebo capsules for that day. We instructed participants to complete the forms immediately after significant interactions, defined as at least 5 minutes of conversation with 1 or more persons. Each day, participants also recorded time of capsule ingestion, and women recorded if they were menstruating. After each treatment phase, participants recompleted the QIDS-SR and the LEIDS-R and were asked about side effects experienced in the past 2 weeks.

### Data Analysis

We excluded social interactions that took place within 3 hours of alcohol ingestion (5.5% of 6141 events). Outcome variables were quarrelsome, agreeable, dominant, and submissive behavior, PA, and NA, positive social cognitions, and negative social cognitions. We considered the within-subjects factor treatment (tryptophan vs placebo), the between-subjects factors order (tryptophan first, placebo first), and their interaction as possible predictors. We did not expect any treatment by gender effects, but to control for the possible effects of gender, we included this factor as a covariate.

To test our hypotheses we used mixed linear modeling with maximum likelihood estimation in R v3.0.2 (www.r-project.org). Statistical significance was set at 0.05. Tukey-Kramer corrections for multiple comparisons were used for analysis of significant interaction effects. Treatment effects are reported using estimated least squares means and SEM. Effect sizes were estimated using Cohen’s *d.* These values represent event-level effects for behaviors and mood and day-level effects for cognitions.

Posthoc analyses were conducted to determine the context in which tryptophan influenced social interactions. Based on previous research (Barker and Lemle, 1987; Moskowitz and Sadikaj, 2012), we created the following categorical contextual variables: location (home, elsewhere), partner sex (male, female), time of interaction (morning, afternoon, evening), and week of treatment phase (week 1, week 2). For the contextual variable relationship status, we created 2 categories: close (friend, romantic partner, or family member) and not close (people at work, acquaintance, or other).

In addition, covariate analyses were performed to examine the extent to which tryptophan-induced changes in social behaviors and cognitions co-occurred with changes in mood. To this end, PA or NA were added as event-level covariates to the models for behavior, and mean PA or NA scores were added as day-level covariates to the models for cognitions.

## Results

There were no significant gender differences in age, QIDS-SR scores, and LEIDS-R scores (Table 1). After completing the study, 31% of the men and 48% of the women were correct in guessing when they were taking tryptophan. These percentages were not significantly different from chance (Table 1).

**Table 1.** Characteristics of Study Participants Expressed in mean (SD) Values

Characteristic	Men (*n* = 13)	Women (*n* = 27)
Age, y	30.4 (13.6)	32.5 (15.2)
QIDS-SR16 score before study	2.8 (1.9)	2.0 (2.0)
QIDS SR16 score after study	2.8 (1.9)	2.3 (1.9)
LEIDS-R score before study	27.4 (13.1)	28.9 (9.3)
LEIDS-R score after study	27.4 (13.8)	27.2 (15.8)
Proband is parent	61 %	48 %
Proband is sibling	31 %	30 %
Proband is child	8 %	22 %

Abbreviations: LEIDS-R, Leiden Index of Depression Severity-Revised; QIDS-SR16, Quick Inventory of Depressive Symptoms–Self-Report 16 items.

### Internal Consistency and Stability across Days of the Behavior Scales

Using the placebo data and otherwise analogous to Moskowitz (1994), we examined inter-item reliability for each of the behavior scales by calculating the Cronbach coefficient α for the 12 items of each scale. Internal consistency was high for all 4 scales (0.75 < α < 0.89).

For stability across days, we constructed scale scores for each placebo day by first calculating the mean of the ipsatized items for each scale for each day and then the Cronbach coefficient α for each scale. Across days, the stability was high for agreeableness (α = 0.79) and quarrelsomeness (α = 0.83) and moderate for dominance (α = 0.68) and submissiveness (α = 0.57). These values are very similar to the values reported by Moskowitz (1994).

#### Planned Analyses at Event Level

Table 2 shows the effects of treatment, order, and the treatment by order interaction on behavior and mood (Table 2).

**Table 2.** F-Values for the Effects of Treatment, Order, and Their Interaction on Behavior and Mood

	Treatment	Order	Treatment × Order
Quarrelsome behaviors	6.11*	4.25*	1.91
Agreeable behaviors	3.04†	0.13	0.95
Dominant behaviors	0.81	4.68*	2.17
Submissive behaviors	0.74	0.40	2.43
Positive affect	10.08**	0.30	5.51*
Negative affect	18.59***	0.57	3.41†

*P < .05, **P < .01, ***P <.001, †*P* < .1.

### Effects of Treatment on Social Behaviors

For quarrelsomeness, the main effects of treatment and order were significant. Quarrelsomeness was higher during tryptophan (*M* = -14.0, SEM = 0.90) than during placebo (*M* = -15.12, SEM = 0.90, *d* = 0.07). Quarrelsomeness was lower in the group that received tryptophan first (*M* = -16.2, SEM = 1.27) than in the group that received placebo first (*M* = -12.96, SEM = 1.12, *d* = 0.65).

For agreeableness, the effects of treatment and order and the treatment by order interaction were not significant. However, agreeable behavior tended to be lower during tryptophan (*M* = 12.99, SEM = 1.15) than during placebo (*M* = 13.98, SEM = 1.15, *d* = 0.05).

For dominant behavior, there was no significant effect of treatment. The main effect of order was significant. Dominant behavior was higher in the group that received tryptophan first (*M* = 9.28, SEM = 1.28) than in the group that received placebo first (*M* = 5.85, SEM = 1.13, *d* = 0.68). The treatment by order interaction was not significant.

For submissive behavior, there were no significant effects of treatment, order, and their interaction.

### Effects of Tryptophan on Mood

For PA, there were significant effects of treatment and of the treatment by order interaction. PA was higher during tryptophan (*M* = 3.36, SEM = 0.18) than during placebo (*M* = 3.21, SEM = 0.18, *t*_5570_ = -4.10, *P* < .001, *d* = 0.11) in participants who received tryptophan second, but not in participants who received tryptophan first (placebo: 3.14 [SEM 0.20]; tryptophan: 3.16 [SEM 0.20], *t*_5569_ = -0.56, *P* = .94, *d* = 0.02).

For NA, there was a significant effect of treatment and a trend for a treatment by order effect. NA was significantly lower during tryptophan (*M* = 0.31, SEM = 0.07) than during placebo (*M* = 0.40, SEM = 0.07, *t*_5556_ = 4.58, *P* < .001, *d* = 0.12) in participants who received tryptophan second, but not in participants who received tryptophan first (placebo: 0.30 [SEM 0.08]; tryptophan: 0.26 [SEM 0.08], *t*_5556_ = 1.66, *P* = .34, *d* = 0.04).

#### Posthoc Analyses at Event Level

We conducted additional analyses to determine whether the (unexpected) increase in quarrelsomeness reported during the tryptophan phase was moderated by context. Quarrelsomeness was the dependent variable, and we entered treatment, order, one of the contextual variables (see Data analysis section), and the 2- and 3-way interactions as predictors. Again, participant gender was added as a covariate. Since agreeableness tended to decrease in the tryptophan phase, we conducted similar analyses for agreeableness.

For both quarrelsomeness and agreeableness, only location (coded as home vs elsewhere) was found to moderate the effects of tryptophan. The percentage of interactions at home was 46.4 % in the placebo phase and 47.4% in the tryptophan phase. Interactions at home primarily involved a romantic partner (37%), followed by a friend (20 %), a parent (15 %), or a child (12 %), with no meaningful differences between the 2 treatment phases.

**Figure 1.**
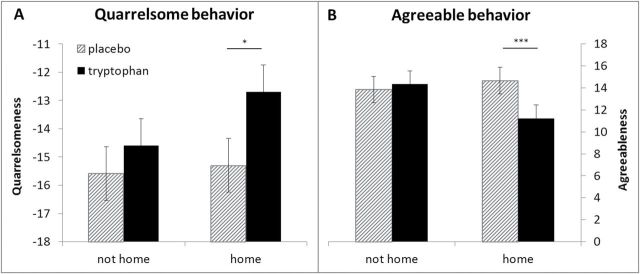
Ipsatized mean scores for quarrelsomeness and agreeableness in interactions at home and away from home during tryptophan and placebo treatment (values are estimated least squares means and SEs).

The treatment by location interaction was significant for quarrelsome behavior (*F*_1,5714_ = 4.05, *P* = .04). Quarrelsomeness was higher during tryptophan than during placebo when participants were at home (*t*_5701_ = -3.00, *P* = .014, *d* = 0.08), but not elsewhere (*t*_5710_ = -0.22, *P* = .99, *d* < 0.01) (Figure 1a).

Similarly, there was a significant treatment by location interaction for agreeable behavior (*F*_1,5714_ = 11.55, *P* < .001). Agreeableness was lower during tryptophan than during placebo when participants were at home (*t*_5702_ = 3.64, *P* = .002, *d* = 0.1) but not elsewhere (*t*_5710_ = -1.12, *P* = .68, *d* = 0.03) (Figure 1b).

For dominant and submissive behavior and for PA and NA, there were no significant interaction effects between treatment and location (all *F*s < 2.25, *P* > .13), nor between treatment and any other contextual variable.

#### Covariate Analyses at Event Level

Quarrelsomeness was negatively associated with PA (*F*_1,4369_ = 107.4, *P* < .001). Similarly, quarrelsomeness was positively associated with NA (*F*_1,5358_ = 90.4, *P* < .001). Nevertheless, the effect of treatment on quarrelsomeness at home remained significant after we controlled for PA or NA (all *t*s > -3.0, *P* < .02).

Agreeableness was positively associated with PA (*F*_1,4439_ = 226.6, *P* < .001). Similarly, agreeableness was negatively associated with NA (*F*_1,5355_ = 69.2, *P* < .001) were associated with higher levels of agreeableness. Nevertheless, the effect of treatment on agreeableness at home remained significant after we controlled for PA or NA (all *t*s > 3.7, *P* < .002).

#### Planned Analyses at Day Level

**Figure 2.**
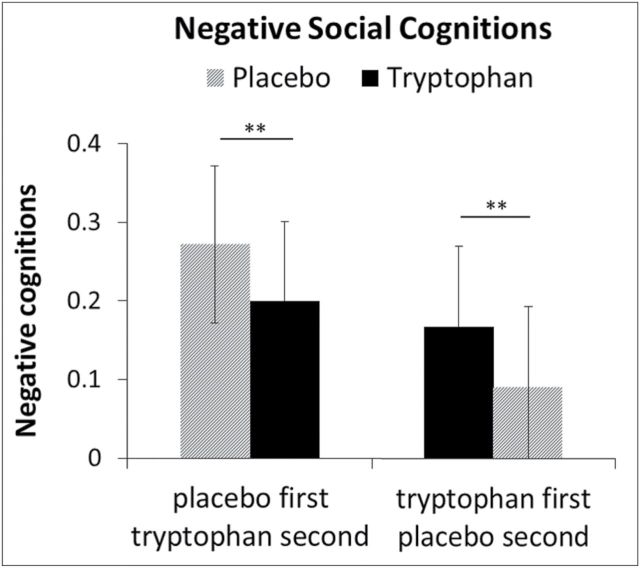
Negative social cognitions during tryptophan and placebo treatment separated by order (values are estimated least squares means and SEs).

For negative social cognitions, there was a significant treatment by order effect (*F*_1,1035_ = 19.4, *P* < .001). Negative social cognitions where lower in the tryptophan phase when tryptophan was given second (*t*_1039_ = 3.26, *P* = .006, *d* = 0.20) and lower under placebo when placebo was given second (*t*_1039_ = -3.0, *P* = .01, *d* = 0.19) (Figure 2).

For positive social cognitions, there were no significant effects of treatment or interactions involving treatment (all *F*s < 2.25, *P* > .14).

#### Covariate Analyses at Day Level

The effect of the treatment by order interaction on negative cognitions remained significant when we controlled for PA, NA, for quarrelsomeness or agreeableness in general, or for quarrelsomeness or agreeableness at home (all *F*s > 12.0, *P* < .001).

## Discussion

We studied the effects of tryptophan on the interpersonal encounters and social cognitions of individuals at risk for depression. Tryptophan improved mood and, specifically during interactions at home, increased quarrelsome behavior and decreased agreeable behavior. Further, negative social cognitions were lower during tryptophan when tryptophan was given second and lower during placebo when it was given second.

The observed behavioral effects were opposite to the hypothesized effects. Tryptophan was previously found to increase agreeableness and decrease quarrelsomeness in individuals with high trait quarrelsomeness (aan het Rot et al., 2006), another population considered at risk for depression. Comparatively, however, levels of quarrelsomeness during tryptophan treatment were significantly lower in our study than levels of quarrelsomeness during placebo treatment in the previous study (but see aan het Rot et al., 2006). Thus, the level to which tryptophan increased quarrelsome behavior in the FH+ individuals was not as high as the baseline level of quarrelsome behavior in the irritable people. This suggests that FH+ individuals may not be as comparable with irritable people as we thought in terms of their social functioning, even though both groups are at elevated risk for MDD.

Given the unexpected behavioral findings, we examined contextual moderators of the effects of tryptophan on quarrelsomeness and agreeableness. We found that the increase in quarrelsomeness and decrease in agreeableness observed during tryptophan treatment only occurred during interactions at home. As the types of interaction partner at home (e.g., romantic partner) were similar for both treatment phases, our behavioral findings cannot be attributed to a phase difference in interaction partners. Moreover, the effects of tryptophan on quarrelsomeness and agreeableness at home were independent of concurrent levels of dominant behavior, submissive behavior, PA, and NA.

As quarrelsomeness often occurs in response to contextual cues, it can be considered a mild form of reactive aggression (Moskowitz, 2010). Laboratory models of aggression in rodents suggest that normal adaptive reactive aggression may be aimed at increasing territorial control and social status and that this type of aggression is positively associated with activity of brain serotonin neurons (de Boer et al., 2009). Therefore, the observed tryptophan-induced increase in quarrelsomeness may have helped the participating FH+ individuals to increase control over their social environment. The fact that behavior changed only at home is consistent with a tendency to be less polite and more critical towards others in familiar situations than in unfamiliar situations (Barker and Lemle, 1987).

Tryptophan supplementation improved mood. This is in line with the previous study by aan het Rot et al. (2006) and with research showing that prolonged increases in brain serotonin induced by antidepressants can improve mood in healthy individuals (Serretti et al., 2010). One other study assessed the effects of prolonged increases in serotonin in FH+ individuals and found no effect on mood (Knorr et al., 2012). However, mood was measured using the Hamilton Depression Rating Scale, which assesses recent depressive symptoms and may not be sensitive to day-to-day changes in mood. In line with this, we found no effect of tryptophan on depressive symptoms measured using the QIDS-SR.

Tryptophan increased both quarrelsome behavior and mood. This may seem in contradiction to the behavioral findings. Indeed, people usually experience more NA when they are more quarrelsome (Moskowitz and Coté, 1995). Yet the effect of tryptophan on quarrelsomeness at home did not change when we controlled for PA or NA. Thus, tryptophan increased quarrelsome behaviors in different interactions at home than in the interactions at home in which it improved mood. A recent EMA study in people with mild to moderate seasonality reported an increase in quarrelsomeness along with an increase in mood during a bright light intervention (Hsu et al., 2014). The authors suggested that an increase in mood in the majority of social interactions together with a small but significant increase in quarrelsomeness in some interactions may be an explanation for their findings. As bright light is thought to increase brain serotonin (aan het Rot et al., 2008), the results of the study by Hsu et al. (2014) are in line with the present results.

To determine the degree to which the effects of tryptophan might extend beyond the level of individual social interactions, at the end of each day we assessed how people thought of themselves in relation to others. When tryptophan was given second, it reduced negative social cognitions, which was in line with our hypothesis. Previous studies in FH+ individuals have reported a negative bias in emotional processing (van der Veen et al., 2007; Feder et al., 2011). Further, research shows that antidepressant treatment can reduce negative biases in emotion processing (Harmer and Cowen, 2013). Thus, it may be that participants who received tryptophan second initially evaluated their social functioning in a negatively biased way and that tryptophan reduced this bias.

 However, when tryptophan was given first, negative social cognitions were subsequently decreased in the placebo phase. This is not in line with the idea that tryptophan reduces negative cognitive biases. One alternative explanation for the findings is that the level of negative social cognitions decreased over time in both treatment order groups. Nevertheless, the possible effects of tryptophan on negative social cognitions need to be interpreted with caution.

### Strengths and Limitations

To our knowledge, this is the first study to assess the effects of increased brain serotonin on daily social functioning in FH+ individuals. As we studied the social interactions that occurred in real life, the study has high ecological validity.

We used a Dutch translation of the SBI. The original English version has been extensively validated (Moskowitz, 1994; Moskowitz and Sadikaj, 2012), and we previously obtained evidence for the construct validity of the Dutch translation (aan het Rot et al., 2013). Further, the internal consistency and reliability across days of the 4 Dutch behavior scales were comparable with those of the English scales, suggesting that the Dutch SBI, like the English SBI, had good psychometric properties.

In spite of these strengths, our study may have been underpowered to examine statistical interaction effects in detail posthoc. Further, the unequal numbers of men and women precluded a comparison of treatment effects between genders. Furthermore, like previous research (Mannie et al., 2007), we included children and siblings of individuals with MDD. Unlike previous studies, however, we also allowed parents to participate (18% of our sample). FH+ parents may have a different MDD risk profile than FH+ siblings and children. In fact, older FH+ individuals in general may be more likely to be resilient to depression than younger individuals. Nevertheless, the results were not moderated by whether participants had a child, sibling, or parent with MDD (data not shown).

Finally, though the family history method we used provides insight in inter-individual variability in familial MDD load (Andreasen et al., 1977), it does not yield MDD diagnoses in relatives. FH+ individuals with multiple family members diagnosed with MDD may respond differently to tryptophan than FH+ individuals with a single affected family member. We were unable to formally test this.

### Future Studies

Our results suggest it may be relevant to examine the effects of increasing brain serotonin on the home interactions of FH+ individuals in more detail. Future studies may focus on the interactions of FH+ individuals with their spouse or with depressed relatives living at home. Interpersonal deficits of depressed individuals tend to be more pronounced in the context of significant relationships (Joiner, 2002; Rehman et al., 2010). This may also be true for FH+ individuals.

As mentioned in the Introduction, Young and colleagues (2014) recently proposed that serotonergic antidepressants in depressed individuals might work by acutely promoting more positive social behavior and that this will gradually result in mood improvement. This idea was largely based on a previous study in individuals with high trait quarrelsomeness, who are also thought to be at risk for MDD (Conner et al., 2003). In this population, tryptophan reduced quarrelsomeness and increased agreeableness (aan het Rot et al., 2006). As we were unable to extend these effects of tryptophan to FH+ individuals in the present study, there are implications for the model proposed by Young et al. (2014). Our results suggest that serotonergic antidepressants might actually increase quarrelsomeness and decrease agreeableness in some MDD patients when they start treatment. While their mood might improve, the effects of social interaction at home would potentially be less desirable and even negatively affect patients’ interpersonal relationships with prolonged treatment. This could be examined in future studies.

In light of the above, a final suggestion for the future would be to conduct a meta-analysis on the effects of tryptophan on everyday interpersonal encounters. This might help elucidate whether, across the 3 EMA studies conducted to date, there exists indeed a group of individuals whose mood improves when they behave in a more agreeable and less quarrelsome way and a group of individuals whose mood improves when they behave in a less agreeable and more quarrelsome way.

### Conclusion

The present study does not provide straightforward evidence that increasing brain serotonin positively influences the interpersonal encounters of individuals with a family history of depression (FH+). However, by increasing quarrelsome behavior and decreasing agreeable behavior at home, tryptophan may provide a way for FH+ individuals to achieve more control. The observed changes in mood and social cognitions are not necessarily in disagreement with this idea.

## Statement of Interest

None.

## Supplementary Material

supplementary information
