# Supplementary material for: The Effects of Tryptophan on Everyday Interpersonal Encounters and Social Cognitions in Individuals with a Family History of Depression
Source: Int J Neuropsychopharmacol. 2015 Mar 23;18(8):pyv012. doi: 10.1093/ijnp/pyv012 (PMC4571634; doi:10.1093/ijnp/pyv012)
Supplement: supplementary information [file ijnp_pyv012_index.html]

Supplementary Data | International Journal of Neuropsychopharmacology

## Supplementary Data

Data files

**Files in this Data Supplement:**

- Supplementary Data - Supplementary Data
