# Supplementary material for: The Effects of Tryptophan on Everyday Interpersonal Encounters and Social Cognitions in Individuals with a Family History of Depression
Source: Int J Neuropsychopharmacol. 2015 Mar 23;18(8):pyv012. doi: 10.1093/ijnp/pyv012 (PMC4571634; doi:10.1093/ijnp/pyv012)
Supplement: supplementary information [file Supplemental_Information.docx]

**Supplemental Information**

**Screening phase**

Probands with a diagnosis of MDD were informed about the study via their health-care provider or via advertisements. They were asked if they had first-degree never-depressed relatives motivated to participate in a study on the effects of tryptophan on social interactions. A total of 206 people responded. Following a brief explanation of the study, 75 probands were interested in receiving further information. We obtained informed consent from 61 affected family members to contact their health-care provider and verify the diagnosis of MDD and absence of a lifetime history of (hypo) mania. Subsequently, we excluded 4 individuals (2 individuals did not meet the criteria for MDD diagnosis; 2 individuals were diagnosed with bipolar disorder) and we were unable to verify the diagnosis with the health-care provider for 3 individuals.

Fifty-eight never-depressed first degree family members were interested in receiving further information. We excluded 6 family members for medical reasons, 5 declined, and 5 were lost to follow up. A total of 42 participants started the study. One participant dropped out after five days and one participant admitted to non-adherence to the study instructions. The results are described for 40 participants (13 men, 27 women).

**Social cognitions**

Cognitions were reported at the end of each day. Six negative items (e.g., “No one understands me”) and six positive items (e.g., “I have a good way with others”) were derived from the Automatic Thoughts Questionnaire (Hollon and Kendall, 1980), the Depression scale of the Cognition Checklist (Beck et al., 1987), the Dysfunctional Attitude Scales (Weissman, 1979), and the Automatic Thoughts Questionnaire-Positive (Ingram and Wisnicki, 1988). Response choices ranged from 0 (not at all) to 6 (all the time). The full list of items is available upon request.

References

Beck AT, Brown G, Steer RA, Eidelson JI, Riskind JH (1987) Differentiating anxiety and depression: A test of the cognitive content-specificity hypothesis. J Abnorm Psychol (US) 96:179-183.

Hollon SD, Kendall PC (1980) Cognitive self-statements in depression: Development of an automatic thoughts questionnaire. Cognitive Therapy and Research (Germany) 4:383-395.

Ingram RE, Wisnicki KS (1988) Assessment of positive automatic cognition. J Consult Clin Psychol (US) 56:898-902.

Weissman AN (1979) The dysfunctional attitude scale: A validation study. Dissertation Abstracts International (US) 40:1389-1390.
